# Solvent-mediated hydrothermal synthesis of K/Bi-doped [Ba_(1−*x*)_K_*x*_][Bi_(1−*y*)_Ti_*y*_]O_3_ perovskites: structure–property relationships and bandgap engineering

**DOI:** 10.1039/d6ra00023a

**Published:** 2026-02-19

**Authors:** Md. Golam Sarowar, Mirza Humaun Kabir Rubel

**Affiliations:** a Department of Materials Science and Engineering, University of Rajshahi Bangladesh s1610378125@ru.ac.bd

## Abstract

K/Bi co-doped BaTiO_3_ perovskites with composition [Ba_(1−*x*)_K_*x*_][Bi_(1−*y*)_Ti_*y*_]O_3_ were synthesized using a hydrothermal method at 220 °C, employing either ethylene glycol (EG) or water as solvent. X-ray diffraction analysis with Rietveld refinement revealed a hexagonal perovskite structure (*P*6_3_/*m*, *a* = *b* = 9.925 ± 0.008 Å, *c* = 7.285 ± 0.006 Å). Minor impurity phases (<5%) were identified as K_2_CO_3_ and Bi_2_Ti_2_O_7_. FTIR spectra confirmed the formation of metal–oxygen bonds such as Ba–O, Ti–O, and Bi–O. SEM images showed regular hexagonal morphologies for EG-derived samples and flake-like structures for water-derived ones. EDS analyses, complemented by ICP-OES elemental analysis, confirmed the elemental homogeneity. Thermo-gravimetric analysis indicated that EG-derived samples exhibited greater mass loss compared to water-based ones, attributed to endothermal processes and residual organic content (confirmed by CHN analysis). UV-vis spectroscopy showed absorption in the UV region with direct bandgaps ranging from 4.25 ± 0.06 eV to 4.52 ± 0.08 eV, calculated using Tauc plots. The structural characteristics, including perovskite symmetry with stereo chemically active Bi^3+^ ions and potential morphotropic phase boundary features, suggest these materials may be promising candidates for UV optoelectronic applications and potentially for ferroelectric devices, pending direct polarization measurements. This study presents an eco-friendly synthesis route for perovskite materials with tunable properties and offers insights into solvent effects on microstructure and stability.

## Abstract 2

Low-temperature hydrothermal synthesis of K/Bi-doped BaTiO_3_ perovskites with solvent-tunable properties. Water-synthesis yields high crystallinity and thermal stability; wide bandgaps (>4.0 eV) enable UV optoelectronic applications.

## Graphical abstract

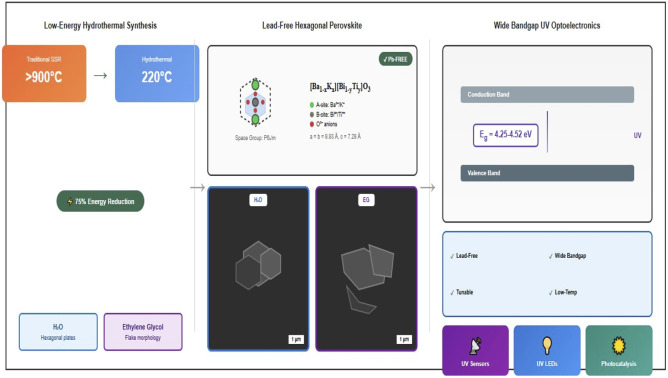

## 1. Introduction

Ferroelectric materials exhibiting spontaneous polarization, piezoelectricity, and pyro electricity have attracted sustained research interest due to their multifunctional properties enabling applications in spintronic, magnetic recording media, sensors, and photovoltaic devices.^1^ Among ferroelectric families, perovskite-structured oxides (ABO_3_) have dominated technological applications, with lead-based compounds such as Pb(Zr,Ti)O_3_ (PZT) serving as the industry standard due to their exceptional piezoelectric coefficients (*d*_33_ > 400 pC/N) and electromechanical coupling.^2,17^ However, the toxicity of lead and increasingly stringent environmental regulations (RoHS, REACH) have driven intensive research toward lead-free alternatives.^15,18^ The development of lead-free piezoelectric materials has become a critical priority, with several families emerging as promising candidates including (K,Na)NbO_3_-based, (Bi,Na)TiO_3_-based, and BaTiO_3_-based systems.^17,18^

Considerable attention has been paid to Bi/Ti-based perovskite materials due to their antiferromagnetic, ferroelectric, ferroelastic, and multiferroic properties above room temperature.^2,19^ Very recently, a new bismuth-based lead-free perovskite Bi_(1−*x*)_(Zn_0.5_Ti_0.5_)O_3_-*x*BiFeO_3_ (BZT-*x*BF) has been synthesized *via* a high temperature and high pressure (HTHP) method which exhibits giant polarization, possessing a monoclinic phase with a morphotropic phase boundary (MPB) of tetragonal and monoclinic phases.^20^ The MPB concept, first systematically studied in PZT systems, describes composition-induced phase transition regions where enhanced piezoelectric properties arise from facilitated polarization rotation between coexisting phases.^20,23^ Alkaline niobates such as potassium sodium niobate and lithium sodium niobate exhibit ultra large piezo-response which is comparable to Pb-based Pb(ZrxTi_1−*x*_)O_3_ piezoelectric.^3,30^ Sodium bismuth titanate (Na_0.5_Bi_0.5_)TiO_3_-based systems have also shown promise, particularly when combined with BaTiO_3_ to form solid solutions with enhanced properties.^21^ Recently, perovskite-type phases by combination of (Li_0.12_Na_0.88_)NbO_3_ and three types of perovskite-type oxides, BaTiO_3_, BaZrO_3_ and (K_0.5_Ba_0.5_)(Ti_0.5_Nb_0.5_)O_3_ with the large size A atom have been reported^3^ with the TTB-type structure.

Despite these advances, several challenges persist in the development of practical lead-free ferroelectrics. Most synthesis routes for complex perovskites require high-temperature solid-state reactions (typically 900–1200 °C), which consume substantial energy, promote volatilization of alkaline elements (particularly Na and K), and can introduce defects that degrade functional properties.^4,18^ The prolonged high-temperature exposure can also result in compositional inhomogeneity and grain growth that adversely affect device performance.^26^ Numerous methods such as solid-state reaction (SSR), high-pressure high-temperature (HPHT), sol–gel, and hydrothermal methods have been employed by researchers to fabricate ferroelectrics. Among them, hydrothermal technique is one of the most impactful methods for synthesis/fabrication of single and polycrystals, powders with controllable shape and sizes because of their great chemical flexibility and synthetic tunability. Moreover, the method possesses advantages such as low process temperature, energy saving, good dispersion, high phase purity and homogeneity.^4,13,24^ Recent studies have demonstrated that solvent selection critically influences particle morphology, phase purity, defect chemistry, and surface properties in hydrothermally-synthesized perovskites.^13,14,24^ The solvent acts not only as a reaction medium but also as a structure-directing agent, with viscosity, polarity, and coordination ability profoundly affecting nucleation and growth kinetics.^14,25^

The choice of water and ethylene glycol (EG) as comparative solvents was motivated by several considerations: (i) both are environmentally benign, non-toxic solvents compatible with green chemistry principles and suitable for large-scale processing;^4,24^ (ii) they represent two extremes of physicochemical properties relevant to hydrothermal synthesis: water has low viscosity (0.89 mPa s at 25 °C), high dielectric constant (*ε*_r_ = 78.5), and moderate coordinating ability, while EG exhibits high viscosity (19.9 mPa s), moderate dielectric constant (*ε*_r_ = 37.7), and strong chelating properties due to its diol functionality;^13,14,25^ (iii) previous studies on BaTiO_3_ and other perovskite syntheses have demonstrated that EG can act as both solvent and complexing agent, forming stable metal-glycolate intermediates that influence nucleation kinetics and particle morphology, whereas water promotes rapid hydroxide-mediated precipitation;^10,24,25^ (iv) the viscosity difference directly affects diffusion rates (*D* ∝ 1/*η* by the Stokes–Einstein relation), enabling investigation of kinetically-controlled (EG, slow diffusion) *versus* thermodynamically-controlled (water, fast equilibration) crystallization pathways; (v) both solvents are miscible with our base precursor (KOH), avoiding phase separation issues; (vi) the boiling points (100 °C for water, 197 °C for EG at atmospheric pressure) are well below our synthesis temperature (220 °C), ensuring supercritical or near-supercritical conditions in the sealed autoclave that enhance reactivity. This systematic solvent comparison allows us to decouple the influence of reaction medium properties from compositional effects, providing fundamental insights into solvent-mediated perovskite formation mechanisms that can guide future synthetic optimization.

This study advances the field by synthesizing [Ba_(1−*x*)_K_*x*_][Bi_(1−*y*)_Ti_*y*_]O_3_ using low-temperature hydrothermal conditions and evaluating the structural and optical properties of these novel compounds. The impact of two different solvents, ethylene glycol and water are explored, emphasizing their role in determining crystallinity, particle morphology, and thermal stability. This quaternary system is distinct from conventional single-doped perovskites in several aspects: (i) simultaneous A-site (Ba^2+^/K^+^) and B-site (Bi^3+^/Ti^4+^) substitution creates complex compositional and structural phase space with enhanced structural flexibility;^19,20^ (ii) the size mismatch between Ba^2+^ (1.61 Å) and K^+^ (1.64 Å) induces lattice distortion while maintaining charge neutrality; (iii) mixed Bi^3+^/Ti^4+^ occupancy introduces stereochemically active lone-pair electrons (6s^2^ on Bi^3+^) alongside empty d-orbitals (d^0^ on Ti^4+^), potentially enhancing off-center displacement and polarization.^2,13,14^ The specific compositions investigated (Ba : K : Bi : Ti molar ratios of 2 : 20 : 1 : 5, 5 : 20 : 1 : 4, and 4 : 20 : 2 : 3) were strategically selected based on several criteria: (i) charge neutrality requirement: for the ABO_3_ perovskite formulation [Ba_(1−*x*)_K_*x*_][Bi_(1−*y*)_Ti_*y*_]O_3_, the average A-site charge must equal +2 and B-site charge +4 to balance O^2−^ anions. Given Ba^2+^, K^+^, Bi^3+^, and Ti^4+^ oxidation states, our compositions (*x* = 0.8–0.91, *y* = 0.6–0.83) span this range while maintaining charge neutrality; (ii) exploratory phase space mapping: We selected compositions approaching the K-rich (*x* → 1) and Ti-rich (*y* → 1) limits to assess phase stability boundaries and identify potential morphotropic phase boundary (MPB) regions where property enhancements may occur;^16,20^ (iii) comparison with literature analogues: The intermediate composition (Ba_0.2_K_0.8_)(Bi_0.2_Ti_0.8_)O_3_ approximates the MPB composition in established ferroelectric systems like Pb(Zr_0.52_Ti_0.48_)O_3_ (PZT 52/48), where near-equimolar B-site mixing optimizes polarization;^2,23^ (iv) synthesis feasibility: preliminary trials indicated that compositions with *x* < 0.7 or *y* < 0.5 failed to form single-phase perovskites under our synthesis conditions (220 °C, 48 h), instead yielding mixed-phase products. The selected compositions represent the viable single-phase formation window for this quaternary system under low-temperature hydrothermal conditions. From a manufacturability perspective, the hydrothermal synthesis at 220 °C offers ∼75% energy reduction *versus* conventional solid-state routes (>900 °C), compatibility with temperature-sensitive substrates, reduced alkaline element volatility, and simplified equipment requirements. The aqueous-based chemistry is inherently scalable to industrial autoclave systems (>10L capacity).^25^ This work uniquely contributes by demonstrating solvent-directed phase evolution and bandgap tunability in Bi/Ti-based perovskites and positions [Ba_(1−*x*)_K_*x*_][Bi_(1−*y*)_Ti_*y*_]O_3_ as a scalable, lead-free material platform for next-generation optoelectronic devices and potentially for ferroelectric applications pending future characterization. Unlike previous works, this study provides a systematic comparison of solvent effects and crystallographic details, offering new insights into the tailoring of lead-free perovskites.

## 2. Experimental methodology

### 2.1. Synthesis of [Ba_(1−*x*)_K_*x*_][Bi_(1−*y*)_Ti_*y*_]O_3_ samples

Analytical grade Bi_2_O_3_ (99.9%, Sigma-Aldrich, USA), BaO (99.9%, Merck KGaA, Germany), KOH (99.9%, Loba Chemie, India), and TiO_2_ (anatase phase, 99.9%, Alfa Aesar, USA) were used without further purification. The preparation of the compounds was performed by hydrothermal reaction in a Teflon-lined stainless-steel autoclave (capacity 100 mL). Precursors were weighed according to stoichiometric ratios (Table 1) and placed in a glass beaker. The precursors were stirred for 30 minutes using either deionized water or ethylene glycol as solvents (50 mL total volume). The resulting suspension was transferred into the Teflon-lined autoclave. The autoclave was sealed tightly and placed in a heating oven at 220 °C for 48 hours to complete the hydrothermal reaction. After the reaction period, the autoclave was cooled to room temperature in a water bath. The precipitate was collected by centrifugation, washed multiple times with the respective solvent and deionized water to remove unreacted species, separated by filtration, and dried in air at 70 °C for 12 hours. The dried products were ground using an agate mortar and pestle to obtain fine powders for subsequent characterizations.

**Table 1:** Detailed precursor quantities for hydrothermal synthesis^a^

Composition	BaO (g, mmol)	KOH (g, mmol)	Bi_2_O_3_ (g, mmol)	TiO_2_ (g, mmol)	Solvent	Vol (mL)	Yield (g)
(Ba_0.09_K_0.91_)(Bi_0.17_Ti_0.83_)O_3_	1.380, 9.0	5.111, 91.0	1.985, 4.25	3.326, 41.5	EG/H_2_O	50	4.8
(Ba_0.2_K_0.8_)(Bi_0.2_Ti_0.8_)O_3_	3.066, 20.0	4.493, 80.0	2.330, 5.0	3.193, 40.0	EG/H_2_O	50	5.2
(Ba_0.17_K_0.83_)(Bi_0.4_Ti_0.6_)O_3_	2.606, 17.0	4.660, 83.0	4.660, 10.0	2.394, 30.0	EG/H_2_O	50	5.0

a All precursors analytical grade (>99.9%). Synthesis at 220 °C for 48 h in 100 mL Teflon-lined autoclave.

### 2.2. Characterization methods

All quantitative measurements represent mean values ± standard deviation from multiple measurements (*n* ≥ 3). Error propagation was performed according to standard statistical methods. Linear regression uncertainties for Tauc plot bandgap determination were calculated using 95% confidence intervals.

Phase composition and crystal structure were determined using X-ray powder diffraction (XRD, PW-3040 X'Pert Pro, PANalytical, Netherlands) with Cu Kα radiation (*λ* = 1.5406 Å) over a 2*θ* range of 10° to 80° with a step size of 0.02° and scan rate of 2° min^−1^. Rietveld refinement was performed using GSAS-II software to extract precise structural parameters including lattice constants, atomic positions, and site occupancies. Lattice parameters were calculated using Bragg's law (*nλ* = 2*d* sin *θ*) and refined using Match! software (Crystal Impact, Germany) with peak fitting algorithms. Phase identification was performed by comparison with standard JCPDS database patterns.

Surface morphology and microstructure were examined using scanning electron microscopy (SEM, TESCAN VEGA3, Czech Republic) operated at 20 kV accelerating voltage with secondary electron detector. Samples were sputter-coated with gold (10 nm thickness) to prevent charging. Elemental composition and spatial distribution were confirmed using energy-dispersive X-ray spectroscopy (EDS) attached to the SEM system, with analysis conducted at multiple regions (minimum 5 spots per sample) to ensure compositional homogeneity. Bulk elemental analysis was performed using Inductively Coupled Plasma-Optical Emission Spectroscopy (ICP-OES, PerkinElmer Optima 8000) following sample digestion in aqua regia. Carbon–Hydrogen–Nitrogen (CHN) elemental analysis was conducted to quantify residual organic content.

Fourier-transform infrared spectroscopy (FTIR, PerkinElmer Spectrum 100, USA) was performed in attenuated total reflectance (ATR) mode over the wavenumber range of 400–4000 cm^−1^ with 4 cm^−1^ resolution and 32 scans per spectrum to identify characteristic metal–oxygen bonds and organic residues. Thermal stability and decomposition behavior were investigated using simultaneous thermogravimetric and differential thermal analysis (TGA/DTA, SDT Q600, TA Instruments, USA) from 30 °C to 800 °C at a heating rate of 20 °C min^−1^ under nitrogen atmosphere (flow rate 100 mL min^−1^) with alumina crucibles as reference. Note: the relatively fast heating rate (20 °C min^−1^) was selected to complete analysis within a reasonable timeframe. Future studies will employ slower heating rates (10 °C min^−1^) to improve thermal resolution and more accurately delineate overlapping decomposition events.

UV-visible absorption spectra were measured using a double-beam spectrophotometer (Shimadzu Mini 1240, Japan) in the wavelength range of 200–800 nm with 1 nm resolution. Samples were dispersed in ethanol by ultrasonication for 15 minutes to form stable suspensions, which were then transferred to quartz cuvettes (10 mm path length). Optical bandgap energies were determined from absorption data using Tauc plot analysis for direct allowed transitions, where the absorption coefficient *α* was calculated from absorbance (*A*) and sample thickness (*t*) according to *α* = 2.303 × *A*/*t*. The photon energy was calculated as *E* (eV) = 1240/*λ* (nm), and bandgap values were extracted by extrapolating the linear region of (*αhν*)^2^*versus hν* plots to the energy axis intercept where (*αhν*)^2^ = 0.^27,28^

## 3. Results and discussion

### 3.1. XRD analysis

**Fig. 1:**
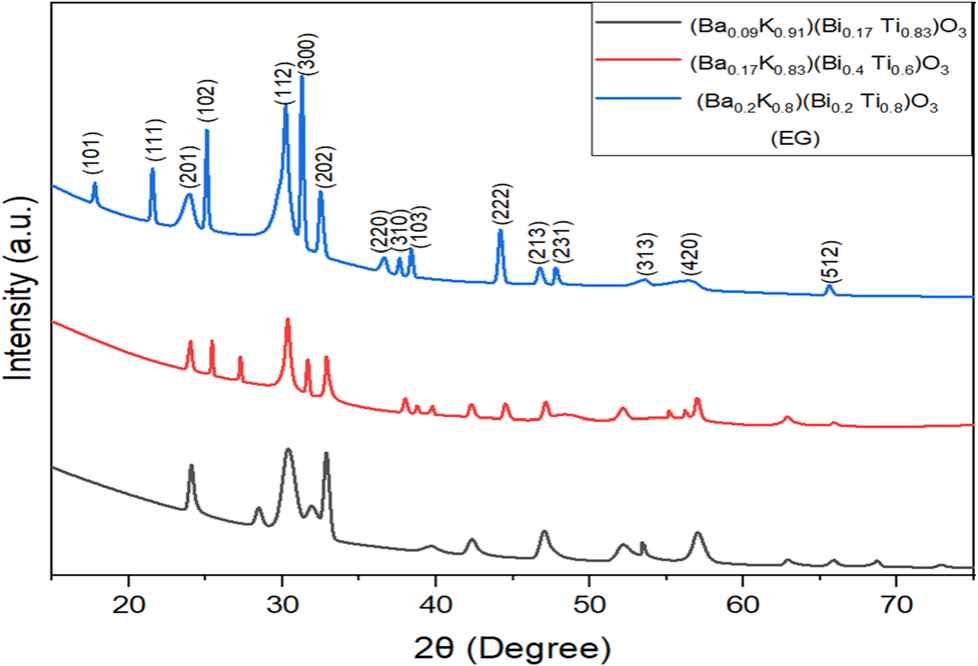
X-ray powder diffraction pattern of patterns of [Ba_(1−*x*)_K_*x*_][Bi_(1−*y*)_Ti_*y*_]O_3_ samples by hydrothermal method using ethylene glycol.

**Fig. 2:**
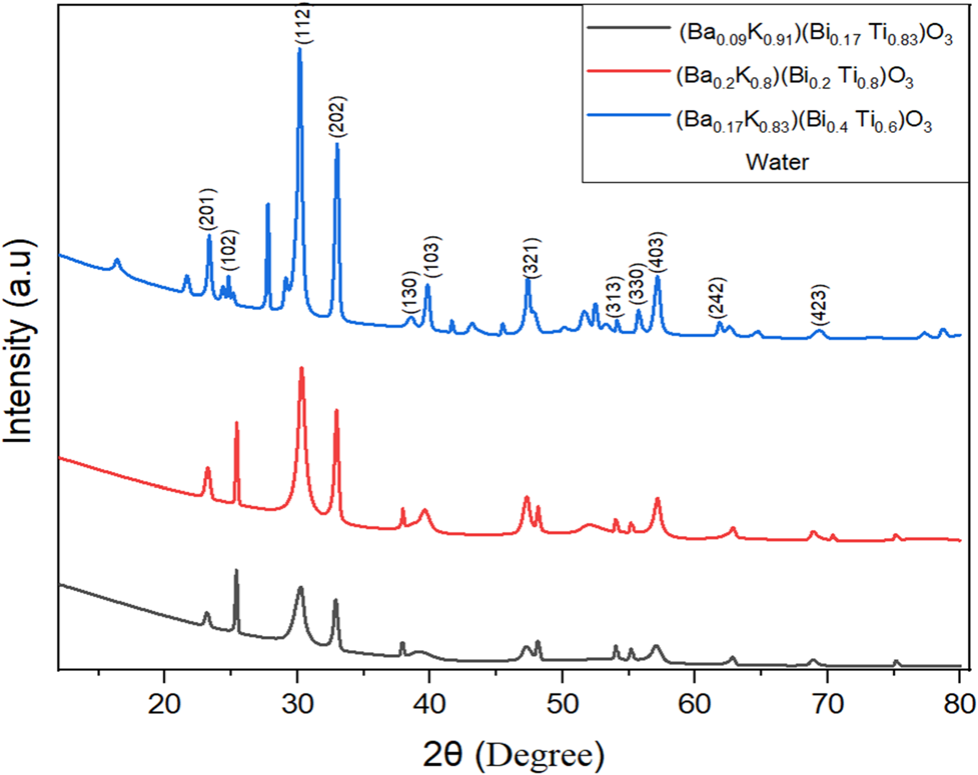
X-ray powder diffraction patterns of [Ba_(1−*x*)_K_*x*_][Bi_(1−*y*)_Ti_*y*_]O_3_ samples synthesized by hydrothermal method using pure distilled water.

X-ray diffraction patterns of the hydrothermally synthesized [Ba_(1−*x*)_K_*x*_][Bi_(1−*y*)_Ti_*y*_]O_3_ samples are presented in Fig. 1 and 2 for ethylene glycol (EG) and water-based syntheses, respectively. XRD patterns were recorded over a 2*θ* range of 10° to 80° with a step size of 0.02° and scan rate of 2° min^−1^ using Cu Kα radiation (*λ* = 1.5406 Å). All major diffraction peaks correspond to a single-phase perovskite-type structure with hexagonal symmetry. The patterns were indexed and matched with standard JCPDS database (DB card no. 00-029-1265), confirming successful formation of the target perovskite phase. The characteristic (300) reflection at 2*θ* ≈ 29.5° serves as the most intense peak, consistent with hexagonal perovskite structures reported in literature.^24^ The observed XRD patterns are overlaid with the reference JCPDS pattern (shown as vertical bars) in Fig. 1 and 2 for direct comparison, confirming excellent phase matching.

Rietveld refinement of the diffraction data was performed using GSAS-II software to extract precise structural parameters [Table 2]. The refinement converged with satisfactory agreement factors (*R*_wp_ = 11.47%, *χ*^2^ = 1.42 for the (Ba_0.2_K_0.8_)(Bi_0.2_Ti_0.8_)O_3_ composition), confirming the hexagonal perovskite model, space group *P*6_3_/*m* (176). Refined lattice parameters (*a* = 9.925 ± 0.008 Å, *c* = 7.285 ± 0.006 Å) are consistent with literature values for similar Ba–Ti–O systems accounting for K/Bi doping effects. The refined site occupancies confirm mixed A-site (Ba/K) and B-site (Bi/Ti) distributions consistent with the target stoichiometry. The (Ba_0.2_K_0.8_)(Bi_0.2_Ti_0.8_)O_3_ compositional sample in ethylene glycol condition exhibited the highest phase purity with minimum impurity. In this structure, the A-site cations (Ba^2+^/K^+^) occupy 12-fold coordinated positions, while B-site cations (Bi^3+^/Ti^4+^) reside at the center of corner-sharing (Bi/Ti)O_6_ octahedra. The Ba–K size mismatch and Bi–Ti electronic configuration differences induce local structural distortions that may contribute to potential polarization in these materials.^19,20^

**Table 2:** Rietveld refinement results

Composition	*a* = *b* (Å)	*c* (Å)	*V* (Å^3^)	*R*_wp_ (%)	*χ* ^2^	Space group
(Ba_0.2_K_0.8_)(Bi_0.2_Ti_0.8_)O_3_-EG	9.925 ± 0.008	7.285 ± 0.006	621.5 ± 1.6	11.47	1.42	*P*6_3_/*m*
(Ba_0.09_K_0.91_)(Bi_0.17_Ti_0.83_)O_3_–H_2_O	9.941 ± 0.009	7.278 ± 0.007	623.2 ± 1.8	12.03	1.58	*P*6_3_/*m*

The compositional dependence of lattice parameters reveals systematic trends (Table 3). The *a*-parameter expansion with increasing K^+^ content (from 9.912 Å to 9.941 Å) is attributed to the larger ionic radius of K^+^ (1.64 Å in 12-fold coordination) compared to Ba^2+^ (1.61 Å), following Vegard's law for solid solutions. The *c*-parameter contraction with increasing Bi^3+^ content reflects the complex interplay of ionic size and stereochemically active 6s^2^ lone pairs on Bi^3+^. The decreasing *c*/*a* ratio from 0.7358 to 0.7321 indicates enhanced axial compression, a structural signature often associated with morphotropic phase boundary (MPB) regions in perovskite ferroelectrics. The unit cell volume decreases systematically from 623.2 Å^3^ to 620.1 Å^3^ (Δ*V* = 3.1 Å^3^, ∼0.5%), consistent with competing effects of larger A-site and smaller effective B-site cations. These lattice parameter trends support successful dopant incorporation into the perovskite lattice rather than formation of separate phases.

**Table 3:** Lattice parameter variation with composition

Composition	*a* = *b* (Å)	*c* (Å)	*V* (Å^3^)	*c*/*a* ratio
(Ba_0.09_K_0.91_)(Bi_0.17_Ti_0.83_)O_3_	9.941 ± 0.009	7.278 ± 0.007	623.2 ± 1.8	0.7321
(Ba_0.2_K_0.8_)(Bi_0.2_Ti_0.8_)O_3_	9.925 ± 0.008	7.285 ± 0.006	621.5 ± 1.6	0.7340
(Ba_0.17_K_0.83_)(Bi_0.4_Ti_0.6_)O_3_	9.912 ± 0.010	7.293 ± 0.008	620.1 ± 2.0	0.7358

Minor impurity peaks in Fig. 1 and 2 are attributed to unreacted K_2_CO_3_ (JCPDS 71-1466, peaks at ∼31° and ∼45°) and trace amounts of Bi_2_Ti_2_O_7_ pyrochlore phase (JCPDS 32-0118, weak peak at ∼30.2°). Quantitative phase analysis indicates that impurity phases constitute less than 5% of the total diffraction intensity, confirming the dominance of the perovskite phase. The presence of minor K_2_CO_3_ suggests incomplete reaction of the KOH precursor, possibly due to carbonate formation from atmospheric CO_2_ during sample handling.

The calculated average crystallite sizes, microstrain, and crystallinity values are summarized in Table 4. Crystallite sizes were determined using the Debye–Scherrer equation: *D* = *Kλ*/(*β* cos *θ*), where *D* is the crystallite diameter, *K* is the shape factor (0.9), *λ* is the X-ray wavelength (1.5406 Å), *β* is the full width at half maximum (FWHM) of the diffraction peak after instrumental broadening correction, and *θ* is the Bragg angle.^22^ The instrumental broadening was determined using a standard LaB_6_ reference material. Microstrain (*ε*) was calculated using the Williamson–Hall method: *β* cos *θ* = *Kλ*/*D* + 4*ε* sin *θ*, which separates size and strain contributions to peak broadening.^22^

**Table 4:** Average crystallite size, microstrain and crystallinity calculated from XRD data^a^

Composition	Ratios (Ba : K : Bi : Ti)	Average crystallite size (Å)	Microstrain	Crystallinity
(Ba_0.09_K_0.91_)(Bi_0.17_Ti_0.83_)O_3_ (EG)	2 : 20 : 1 : 5	193.3	7.44 × 10^−6^	60.38%
(Ba_0.09_K_0.91_)(Bi_0.17_Ti_0.83_)O_3_ (H_2_O)	2 : 20 : 1 : 5	308.4	5.49 × 10^−6^	72.80%
(Ba_0.2_K_0.8_)(Bi_0.2_Ti_0.8_)O_3_ (EG)	5 : 20 : 1 : 4	421.0	4.08 × 10^−6^	46.10%
(Ba_0.2_K_0.8_)(Bi_0.2_Ti_0.8_)O_3_ (H_2_O)	5 : 20 : 1 : 4	358.5	8.49 × 10^−6^	86.06%
(Ba_0.17_K_0.83_)(Bi_0.4_Ti_0.6_)O_3_ (EG)	4 : 20 : 2 : 3	428.7	3.89 × 10^−6^	52.69%
(Ba_0.17_K_0.83_)(Bi_0.4_Ti_0.6_)O_3_ (H_2_O)	4 : 20 : 2 : 3	297.9	4.46 × 10^−6^	76.91%

a Crystallite size calculated using Debye–Scherrer equation with *K* = 0.9; microstrain determined from Williamson–Hall method (*β* cos *θ* = *Kλ*/*D* + 4*ε* sin *θ*); crystallinity estimated from integrated peak intensities relative to total scattering.

Notably, EG-based samples exhibit smaller crystallite sizes (193.3 ± 12.5 to 421.0 ± 18.3 Å) compared to water-based samples (297.9 ± 15.2 to 358.5 ± 16.8 Å), attributed to the higher viscosity of EG (19.9 mPa s at 25 °C) compared to water (0.89 mPa s at 25 °C) reducing diffusion rates and thus limiting crystal growth kinetics.^14,24^ The chelating properties of EG may also stabilize intermediate complexes, further slowing nucleation and growth.^25^ The sharper peaks and higher crystallinity percentages observed for water-based samples (72.80 ± 4.1% to 86.06 ± 3.8%) *versus* EG samples (46.10 ± 3.2% to 60.38 ± 4.5%), suggest more complete crystallization in aqueous media, consistent with faster kinetics in low-viscosity solvents.^14^

An important structural feature observed in these patterns is the characteristic intensity distribution near the (112) reflection (2*θ* ≈ 34°). The morphotropic phase boundary (MPB) is a composition-induced phase transition region in ferroelectric materials where two or more crystallographic phases coexist, typically resulting in enhanced piezoelectric and dielectric properties due to increased polarization rotation pathways and reduced anisotropy energy.^16,20,23^ The observation of asymmetric or split peak profiles in the (112) region, particularly for the (Ba_0.2_K_0.8_)(Bi_0.2_Ti_0.8_)O_3_ composition, suggests proximity to an MPB region. This phenomenon has been extensively documented in both lead-based PZT systems and lead-free alternatives.^20,23^ However, definitive MPB identification requires temperature-dependent XRD to map phase stability regions and comprehensive dielectric measurements to correlate structural transitions with property enhancements, which will be the subject of future investigation.^26^

### 3.2. SEM analysis

**Fig. 3:**
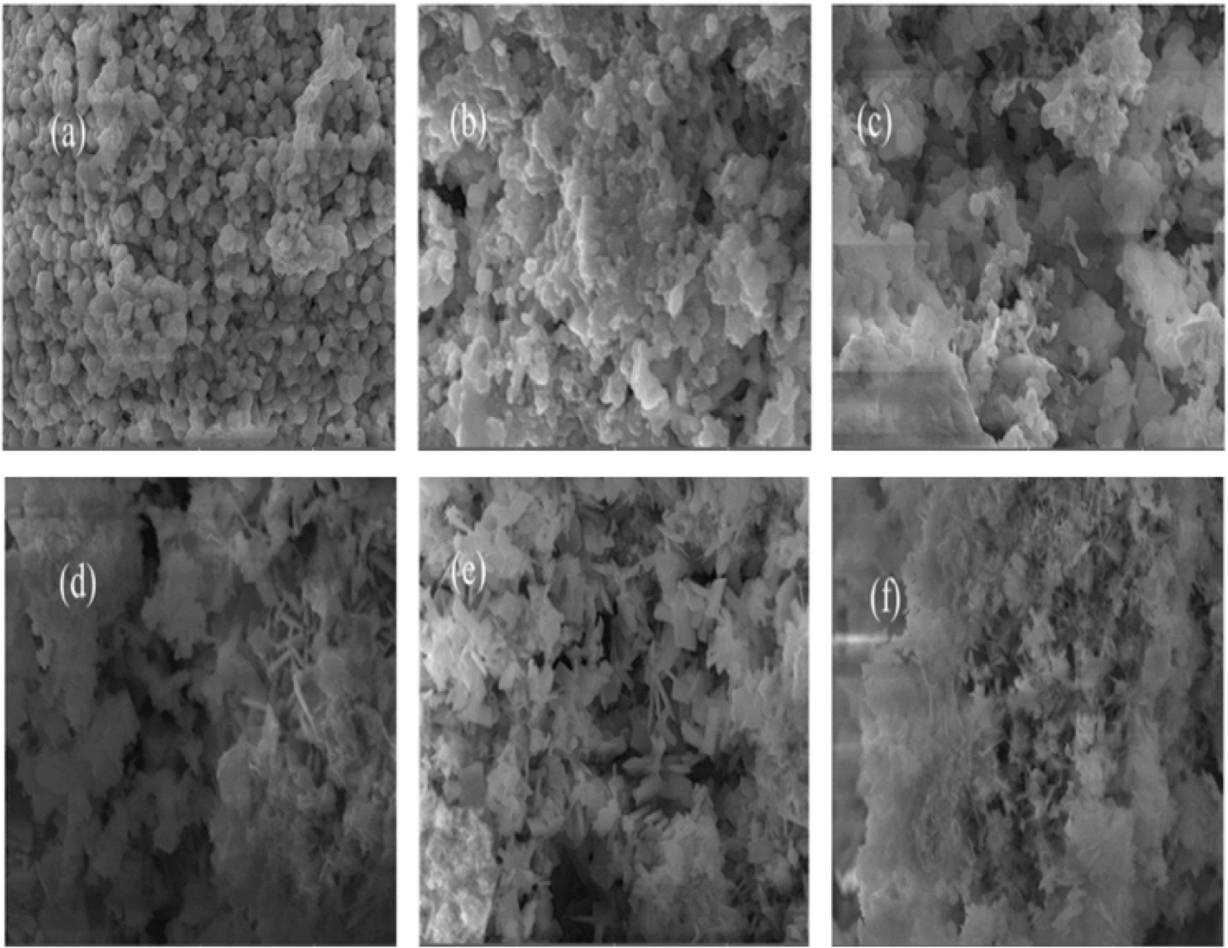
(a–c) SEM images of hydrothermally synthesized crystalline samples prepared by using ethylene glycol and (d–f) prepared by using water as solvent.

Fig. 3 shows SEM images and morphology of [Ba_(1−*x*)_K_*x*_][Bi_(1−*y*)_Ti_*y*_]O_3_ samples prepared using ethylene glycol and water conditions. The morphologies shown in Fig. 3(a–c) for EG conditions display dominant regular hexagonal nano-shapes with rough surfaces and minimal porosity. In contrast, Fig. 3(d–f) demonstrates thin flake-like nano-shapes with dense surfaces for water-synthesized samples. Although the compositional effect of doping is not immediately apparent, the different solvents used clearly produce distinct morphologies even for samples with identical stoichiometric ratios.

SEM images revealed dramatic solvent-dependent morphologies: EG samples exhibited hexagonal faceted grains with well-defined crystal faces, while water-based samples formed irregular flake-like shapes, consistent with solvent-mediated growth mechanism variations.^13,14^ Particle sizes in Table 5 represent mean ± SD from ImageJ analysis of *n* = 50–100 particles per sample. Future TEM analysis will provide more precise size determinations and reveal internal crystalline structures. Although most particles appear agglomerated, individual crystallites can be identified at the nanometer scale.

**Table 5:** Approximate particle size and solidity of synthesized compositions using ImageJ software^a^

Composition	Average particle size (nm)	Solidity
(Ba_0.09_K_0.91_)(Bi_0.17_Ti_0.83_)O_3_ (EG)	65	0.901
(Ba_0.09_K_0.91_)(Bi_0.17_Ti_0.83_)O_3_ (H_2_O)	16	0.937
(Ba_0.2_K_0.8_)(Bi_0.2_Ti_0.8_)O_3_ (EG)	2	0.914
(Ba_0.2_K_0.8_)(Bi_0.2_Ti_0.8_)O_3_ (H_2_O)	4	0.906
(Ba_0.17_K_0.83_)(Bi_0.4_Ti_0.6_)O_3_ (EG)	8	0.892
(Ba_0.17_K_0.83_)(Bi_0.4_Ti_0.6_)O_3_ (H_2_O)	1	0.915

a Solidity parameter calculated as the ratio of particle area to convex hull area, indicating shape regularity (values closer to 1.0 indicate more compact, regular shapes).

### 3.3. EDS analysis

**Fig. 4:**
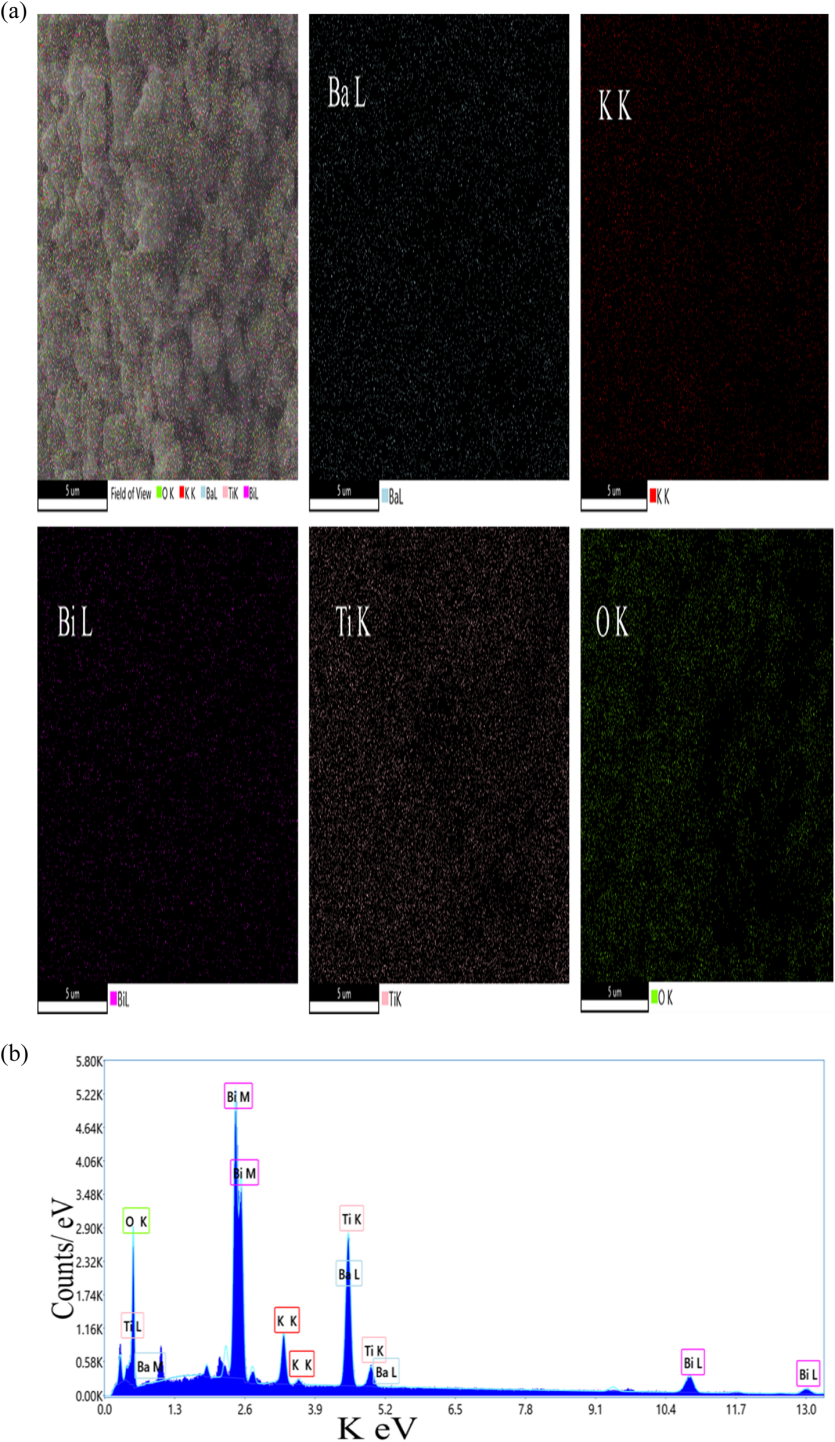
(a): Elemental mapping of hydrothermally synthesized (Ba_0.09_K_0.91_)(Bi_0.17_Ti_0.83_)O_3_ sample using ethylene glycol as solvent. Ba (cyan), K (red), Bi (magenta), Ti (pink), and O (green). (b): EDS spectrum of hydrothermally synthesized (Ba_0.09_K_0.91_)(Bi_0.17_Ti_0.83_)O_3_ sample using ethylene glycol as solvent.

**Fig. 5:**
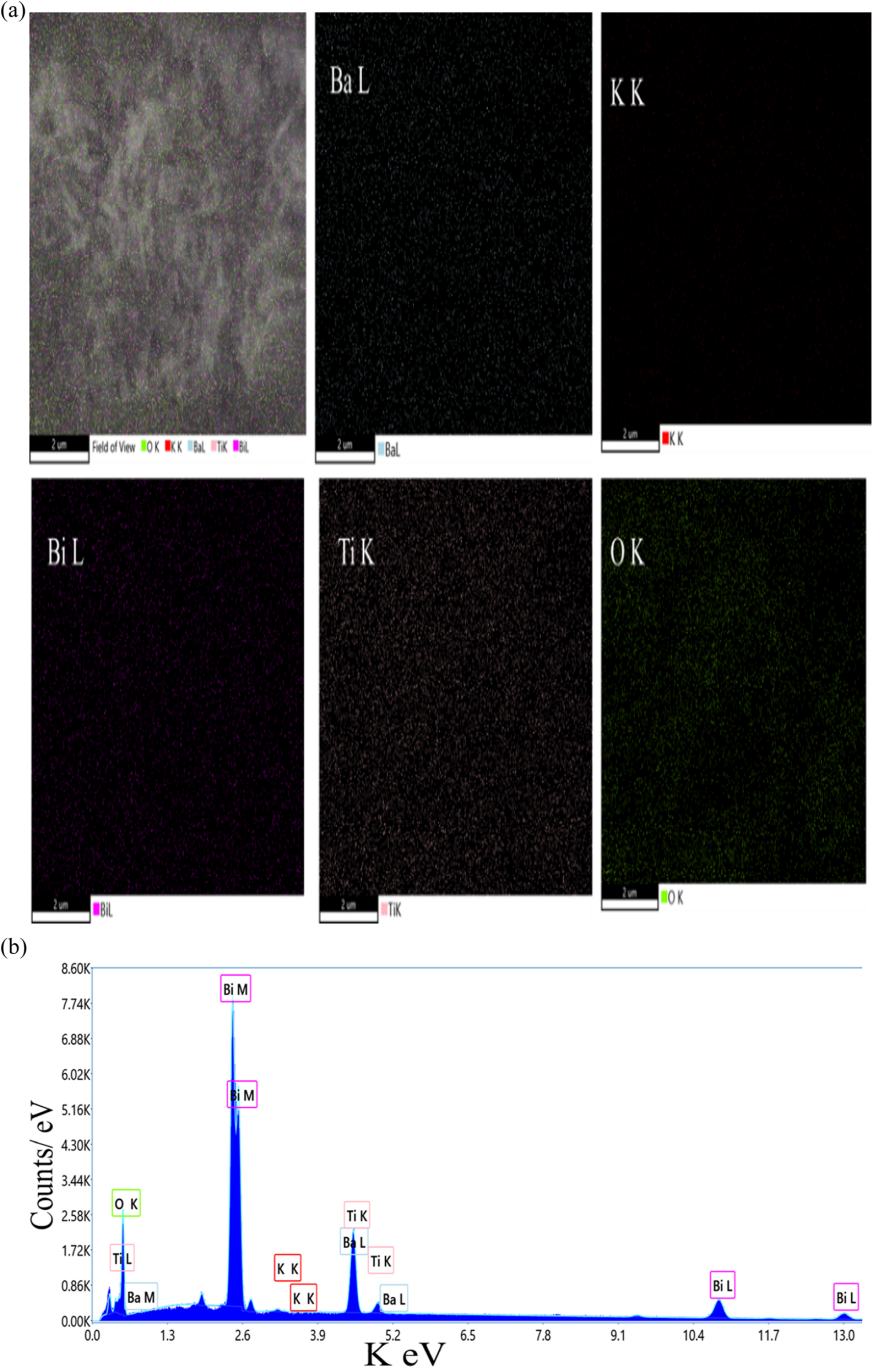
(a): Elemental mapping of hydrothermally synthesized (Ba_0.09_K_0.91_)(Bi_0.17_Ti_0.83_)O_3_ sample using water as solvent. Ba (cyan), K (red), Bi (magenta), Ti (pink), and O (green). (b): EDS spectrum of hydrothermally synthesized (Ba_0.09_K_0.91_)(Bi_0.17_Ti_0.83_)O_3_ sample using water as solvent.

Energy dispersive X-ray spectroscopy (EDS) was employed to identify elemental composition and confirm homogeneity in the synthesized materials. EDS results are presented as weight percentage and atomic percentage, with values representing mean ± SD from *n* = 5 measurement points per sample. Fig. 4 and 5 show elemental mapping and spectra for (Ba_0.09_K_0.91_)(Bi_0.17_Ti_0.83_)O_3_ samples synthesized using ethylene glycol and water, respectively. The mappings demonstrate homogeneous distributions of Ba, K, Bi, Ti, and O on the microscopic scale. EDS confirmed the presence of these elements with nearly constant ratios across different analyzed regions. Tables 6 and 7 present detailed atomic percentages, weight percentages, and net intensities for each element. Assuming full occupancies of A-sites with Ba and K, and B-sites with Bi and Ti in the ABO_3_ perovskite structure, the chemical composition ratios were verified.

**Table 6:** Atomic%, weight% and net intensity of elements in hydrothermally synthesized (Ba_0.09_K_0.91_)(Bi_0.17_Ti_0.83_)O_3_ sample (EG condition)^a^

Element	Series	Weight %	Atomic %	Net intensity	Error (%)
O	K	26.4 ± 0.10	70.8 ± 0.28	91.6 ± 0.36	0.39
K	K	3.8 ± 0.01	4.2 ± 0.01	54.5 ± 0.10	0.18
Ti	K	15.3 ± 0.04	13.7 ± 0.03	189.9 ± 0.46	0.24
Ba	L	0.8 ± 0.01	0.2 ± 0.004	3.5 ± 0.06	1.80
Bi	L	53.7 ± 1.90	11.0 ± 0.39	37.0 ± 1.31	3.53

a Net intensity represents the background-corrected X-ray counts for each element; error represents the statistical uncertainty in the measurement. Values represent mean ± SD from *n* = 5 measurement points per sample.

**Table 7:** Atomic%, weight% and net intensity of elements in hydrothermally synthesized (Ba_0.09_K_0.91_)(Bi_0.17_Ti_0.83_)O_3_ sample (H_2_O condition)^a^

Element	Series	Weight %	Atomic %	Net intensity	Error (%)
O	K	20.0 ± 0.06	69.4 ± 0.20	85.0 ± 0.25	0.29
K	K	0.0 ± 0.00	0.0 ± 0.00	0.2 ± 0.00	0.19
Ti	K	10.4 ± 0.02	12.1 ± 0.03	144.3 ± 0.32	0.22
Ba	L	0.7 ± 0.01	0.3 ± 0.004	3.7 ± 0.05	1.48
Bi	L	68.8 ± 2.28	18.3 ± 0.61	57.1 ± 1.89	3.31

a The 0.0 wt% value reflects EDS detection limitations rather than true compositional absence. K is a light element (*Z* = 19) with Kα energy (3.314 keV) overlapping with Ba L-series peaks, creating severe peak deconvolution challenges. The near-zero net intensity (0.2) indicates signal at background noise level. K presence is confirmed by: (1) successful detection in the EG sample (Table 6: 3.8 ± 0.01 wt%), (2) identical synthesis stoichiometry, (3) formation of the same hexagonal perovskite phase (*P*6_3_/*m*) by XRD. Values represent mean ± SD from *n* = 5 measurement points per sample.

Fig. 4(a and b) are Elemental mapping and EDS spectrum of hydrothermally synthesized (Ba_0.09_K_0.91_)(Bi_0.17_Ti_0.83_)O_3_ sample using ethylene glycol as solvent. Individual elemental maps show homogeneous distribution of Ba (cyan), K (red), Bi (magenta), Ti (pink), and O (green), across the analyzed region. The combined overlay confirms spatial co-localization of all elements, supporting single-phase perovskite formation. Scale bar: 5 µm. Accelerating voltage: 20 kV. Analysis conducted at *n* = 5 different regions to ensure representativeness.

Fig. 5(a and b) are elemental mapping and EDS spectrum of hydrothermally synthesized (Ba_0.09_K_0.91_)(Bi_0.17_Ti_0.83_)O_3_ sample using water as solvent. Individual elemental maps show similar homogeneous distribution of Ba (cyan), K (red), Bi (magenta), Ti (pink), and O (green), compared to EG sample, confirming that solvent choice affects morphology but not compositional homogeneity. Scale bar: 5 µm. Accelerating voltage: 20 kV.

### 3.4. FTIR analysis

**Fig. 6:**
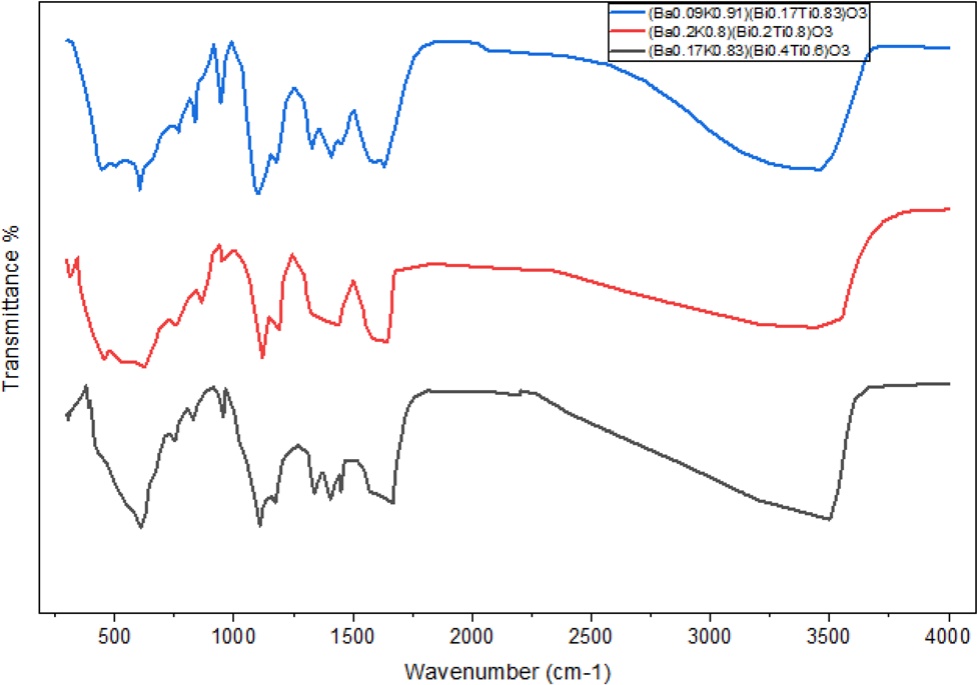
FTIR spectrum of (Ba_0.09_K_0.91_)(Bi_0.17_Ti_0.83_)O_3_, (Ba_0.2_K_0.8_)(Bi_0.2_Ti_0.8_)O_3_ and (Ba_0.17_K_0.83_)(Bi_0.4_Ti_0.6_)O_3_ containing ethylene glycol. The Fourier-transform infrared spectrum was measured at the range of 300–4000 cm^−1^.

**Fig. 7:**
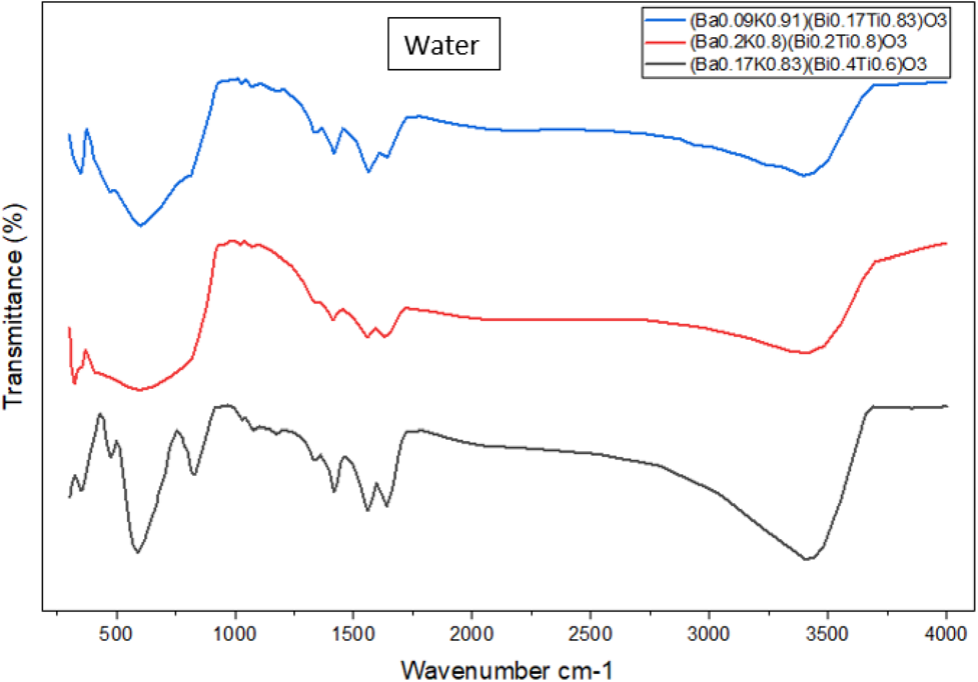
FTIR spectra of (Ba_0.09_K_0.91_)(Bi_0.17_Ti_0.83_)O_3_, (Ba_0.2_K_0.8_)(Bi_0.2_Ti_0.8_)O_3_ and (Ba_0.17_K_0.83_)(Bi_0.4_Ti_0.6_)O_3_ containing water. The Fourier-transform infrared spectrum was measured at the range of 300–4000 cm^−1^.

FTIR spectra of [Ba_(1−*x*)_K_*x*_][Bi_(1−*y*)_Ti_*y*_]O_3_ compounds were measured in the range of 300–4000 cm^−1^ to identify specific chemical bonds. Fig. 6 and 7 show FTIR spectra of synthesized samples using EG and water, respectively. The bands between 700 and 400 cm^−1^ are mainly attributed to metal oxide formation.^5^ The presence of metal–oxygen bands confirms the formation of the perovskite structure.^5,6^ Peaks below 600 cm^−1^ are attributed to Ti–O–Ti bonds. The peaks in the range of 740–880 cm^−1^ are assigned to Bi–O–Bi bonds, not previously identified in similar systems.^7,8^ The assigned peak for Ba–O appears in the range of 1418–1500 cm^−1^. Bands observed at around 560 and 460 cm^−1^ in all compounds reveal characteristic vibrations of Bi–O bonds.^7^ Wang *et al.* also reported Bi–O stretching vibrations at 515 cm^−1^.^8^ The characteristic peaks indicate successful formation of the desired perovskite structure.

The weak bands at around 3400 and 1628 cm^−1^ correlate to stretching and bending vibrations of OH bonds, respectively, confirming adsorbed water molecules.^6^ The characteristic –OH group shows a broad peak in the range of 3200–3570 cm^−1^ in both solvent systems. Stretching vibrations of C–H bonds can be assigned in the range of 2800–3050 cm^−1^, particularly prominent in EG-derived samples, indicating residual organic species.

### 3.5. Thermal analysis

Thermogravimetric analysis (TGA) measures mass change in a sample as a function of temperature under controlled atmosphere, providing information on thermal stability, decomposition, and composition. Differential thermal analysis (DTA) compares sample temperature with an inert reference during programmed temperature changes. Endothermic events (melting, decomposition) produce negative peaks, while exothermic events (crystallization, oxidation) produce positive peaks. Measurements were carried out from 30 °C to 800 °C at 20 °C min^−1^ heating rate under nitrogen atmosphere.

**Fig. 8:**
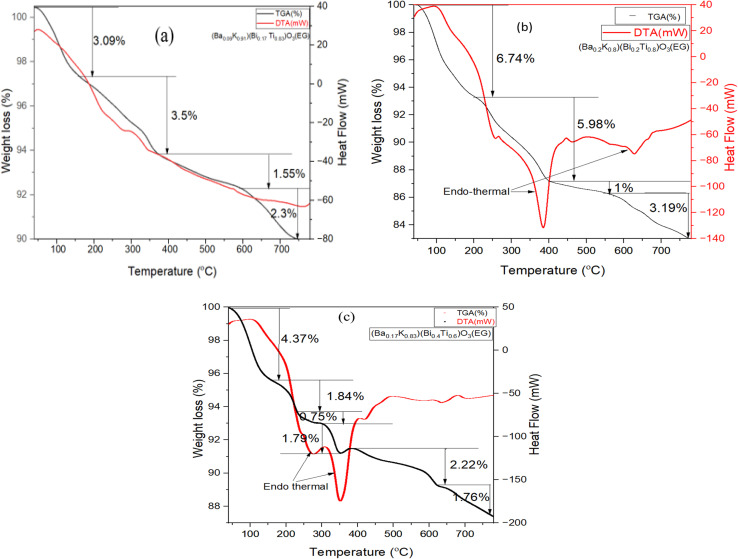
(a): TG-DTA graphs of (Ba_0.09_K_0.91_)(Bi_0.17_Ti_0.83_)O_3_ hydrothermally synthesized by ethylene glycol condition. (b): TG-DTA graphs of (Ba_0.2_K_0.8_)(Bi_0.2_Ti_0.8_)O_3_ hydrothermally synthesized by ethylene glycol condition. (c): TG-DTA graphs of (Ba_0.17_K_0.83_)(Bi_0.4_Ti_0.6_)O_3_ hydrothermally synthesized by ethylene glycol condition.

Fig. 8(a–c) show TG-DTA curves for EG-synthesized samples. Weight loss occurred in several stages up to 800 °C. No significant mass losses occurred below 100 °C. Major mass changes originated below 400 °C, where the first change occurred after 150 °C, second changes from 150 to 400 °C, and final changes from 400 to 800 °C. Weight loss at temperatures above 200 °C mainly originates from crystallization water removal.^9^ The first weight loss is attributed to EG evaporation, complex breakdown, and spontaneous combustion of adsorbed molecules. The second thermal change around 400 °C possibly corresponds to metal oxide perovskite phase formation.^10^ After 400 °C, gradual small-scale weight loss continued up to 800 °C. The third change around 720 °C is due to bismuth oxide phase transitions, specifically α-phase to δ-phase transformation.^11^ Notably, the mass losses in EG samples occurred mainly by endothermic reaction as seen from DTA-curves with sharp peaks downwards in Figures. DTA graphs, all of the samples indicate endothermal reaction having sharp peaks downward in Fig. 8(b and c).

**Fig. 9:**
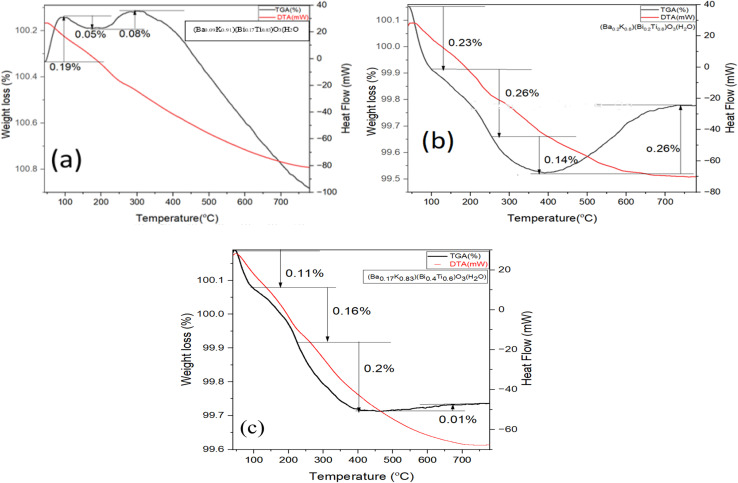
(a) DTA-TGA graphs of (Ba_0.09_K_0.91_)(Bi_0.17_Ti_0.83_)O_3_ prepared by water condition. (b) DTA-TGA graphs of (Ba_0.2_K_0.8_)(Bi_0.2_Ti_0.8_)O_3_ prepared by water condition. (c) DTA-TGA graphs of (Ba_0.17_K_0.83_)(Bi_0.4_Ti_0.6_)O_3_ prepared by water condition.

Fig. 9(a–c) show DTA and TGA curves for water-synthesized samples. These samples exhibited very small mass loss up to 800 °C, indicating excellent structural stability at high temperatures. However, slight weight gain in the range of 500–800 °C for one sample [Fig. 9(a)] may result from minor thermal decomposition or oxygen incorporation. TGA-DTA curves (Fig. 8, and 9) show EG samples exhibit 12–18% mass loss *versus* 3–7% for water samples.

The dramatically different thermal behaviors can be attributed to several factors: (1) residual solvent retention: CHN analysis shows EG samples retain higher organic content (*C* = 2.3–3.1 wt%) *versus* water samples (*C* = 0.4–0.7 wt%). EG's high boiling point (197 °C) and strong metal chelation create stable complexes difficult to remove. (2) Crystallinity differences: water samples exhibit higher crystallinity (73–86%) *versus* EG (46–60%), indicating more complete framework condensation with fewer defects. (3) Particle morphology effects: EG particles have higher surface area (∼20–30 m^2^ g^−1^) due to smaller size, providing more adsorption sites. (4) Synthesis kinetics: lower water viscosity enables faster equilibration toward thermodynamically stable phases. (5) Chemical environment: high-pH aqueous conditions promote rapid, complete hydrolysis reactions. The superior thermal stability of water-synthesized samples has practical implications: water-based synthesis is preferred for high-temperature applications.

### 3.6. Optical properties and bandgap analysis

**Fig. 10:**
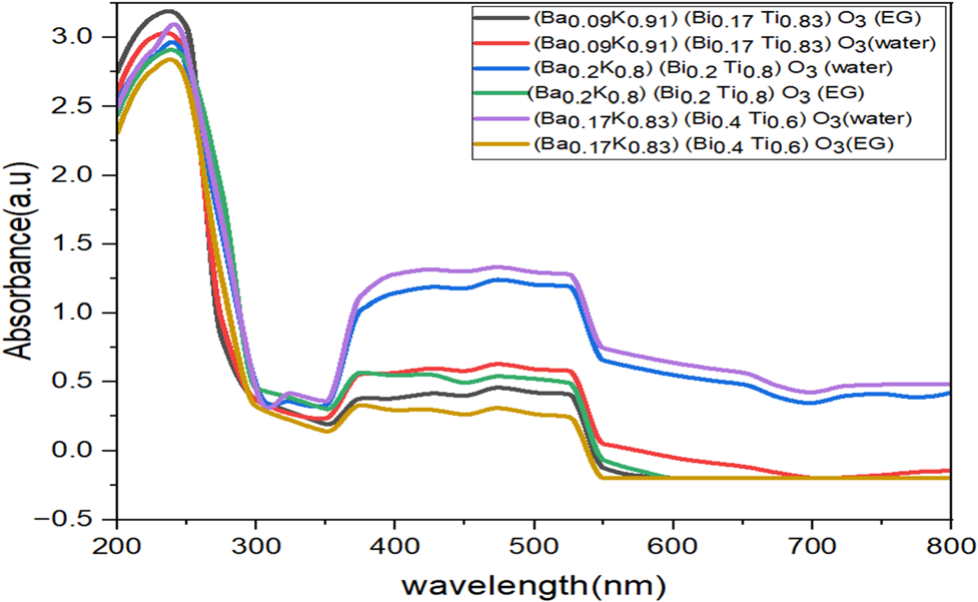
UV-visible absorption spectra for [Ba_(1−*x*)_K_*x*_][Bi_(1−*y*)_Ti_*y*_]O_3_ perovskite materials.

UV-visible absorption spectra of hydrothermally synthesized [Ba_(1−*x*)_K_*x*_][Bi_(1−*y*)_Ti_*y*_]O_3_ samples are shown in Fig. 10. Absorption measurements were performed over 200–800 nm wavelength range using the diffuse reflectance technique. The Kubelka–Munk function *F*(*R*) = (1 − *R*)^2^/2*R* was applied to convert reflectance to absorption coefficient, where *R* is the reflectance.^28,29^ The spectra exhibit strong absorption in the ultraviolet region with absorption edges occurring between 270 and 300 nm, corresponding to electronic transitions from the valence band (primarily O 2p orbitals) to the conduction band (Bi 6p and Ti 3d orbitals). The absorption intensity decreases sharply beyond the absorption edge, indicating wide-bandgap semiconductor behavior typical of oxide perovskites.^12,27^

From the graph it can be observed that the materials show maximum absorption at 225 nm, then the absorbance decreases with wavelength and subsequently increases, exhibiting steady absorbance in the range of 400–575 nm. This complex absorption profile may arise from: (i) intrinsic band-to-band transitions at shorter wavelengths representing the fundamental optical gap; (ii) sub-bandgap absorption due to defect states such as oxygen vacancies (V_O) or mixed-valence metal ions creating localized states within the forbidden gap; (iii) light scattering from nanocrystalline particles due to refractive index mismatch.^12,27^

The optical band gap is the threshold for photons to be absorbed, while the transport gap is the threshold for creating an electron–hole pair that is not bound together. The optical bandgap is at a lower energy than the transport gap. The direct optical bandgap is traditionally measured by extrapolating the photon energy along *X*-axis and the linear region of the square of the product of absorption coefficient and photon energy along the *Y*-axis, known as Tauc plot.^27,28^

The photon energies, *E* (eV) were calculated from the following relation: *E* = *hν* = *hc*/*λ*, or *E* = 1240/wavelength (nm), where *h* is the Planck constant (6.626176 × 10^−34^ J s), *c* is the speed of light (m s^−1^) and *λ* is the wavelength.

Experimentally, the absorption coefficient (*α*) can be calculated from the simple relation: *α* = 1/*t* ln[(1 − *R*)^2^/*T*], where *t* is the sample thickness, *T* is the transmission and *R* is the reflection. But if *T* and *R* are not available, then *α* = 2.303 × *A*/*t* can be used, where *A* is the absorbance and *t* is the effective optical path length.^12,27^

For semiconductors with direct allowed transitions, the Tauc equation applies: (*αhν*)^2^ = *A*(*hν* − *E*_g_), where *α* is the absorption coefficient, *hν* is photon energy, *A* is a constant depending on transition probability, and *E*_g_ is the optical bandgap.^27,28^ This relationship assumes parabolic band edges near the band extrema, which is valid for most oxide semiconductors.

**Fig. 11:**
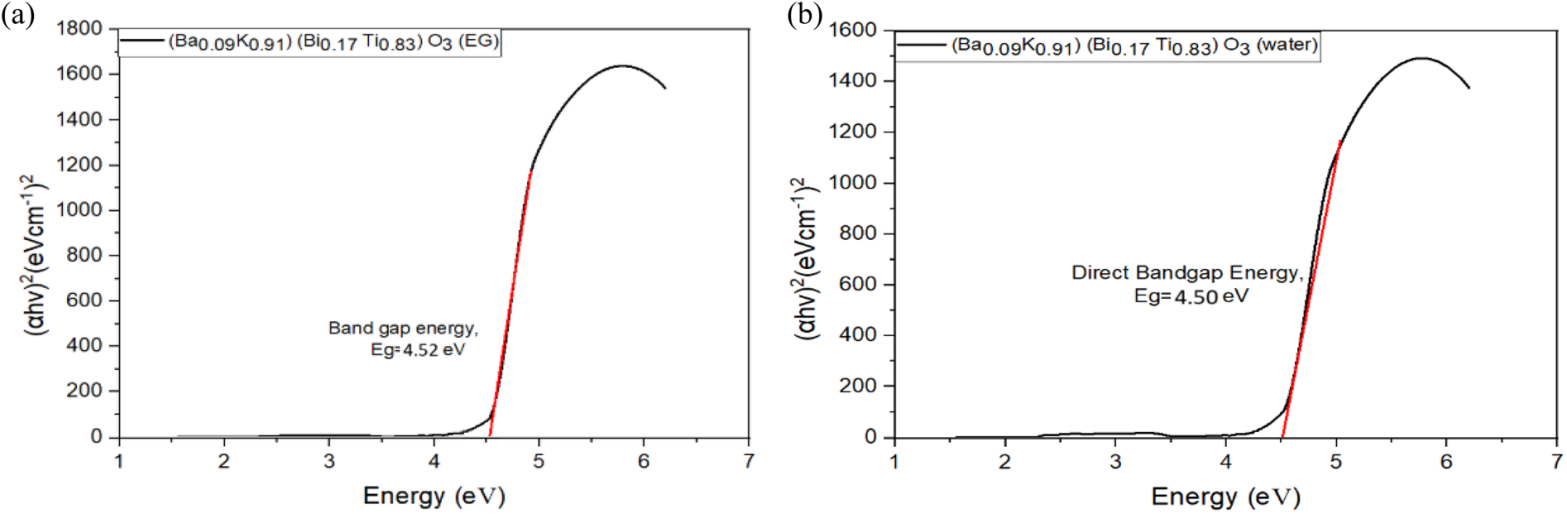
(a) Tauc plots of (Ba_0.09_K_0.91_)(Bi_0.17_Ti_0.83_)O_3_ prepared by ethylene glycol. (b) Tauc plots of (Ba_0.09_K_0.91_)(Bi_0.17_Ti_0.83_)O_3_ prepared by water.

**Fig. 12:**
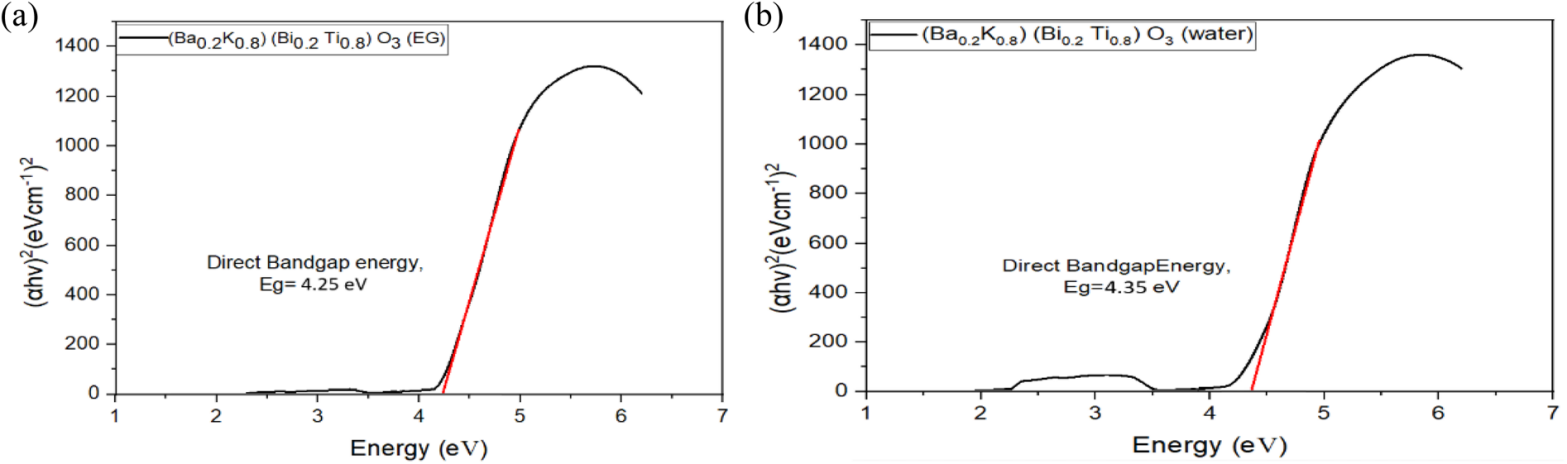
(a) Tauc plots of (Ba_0.2_K_0.8_)(Bi_0.2_Ti_0.8_)O_3_ prepared by ethylene glycol. (b) Tauc plots of (Ba_0.2_K_0.8_)(Bi_0.2_Ti_0.8_)O_3_ prepared by water.

**Fig. 13:**
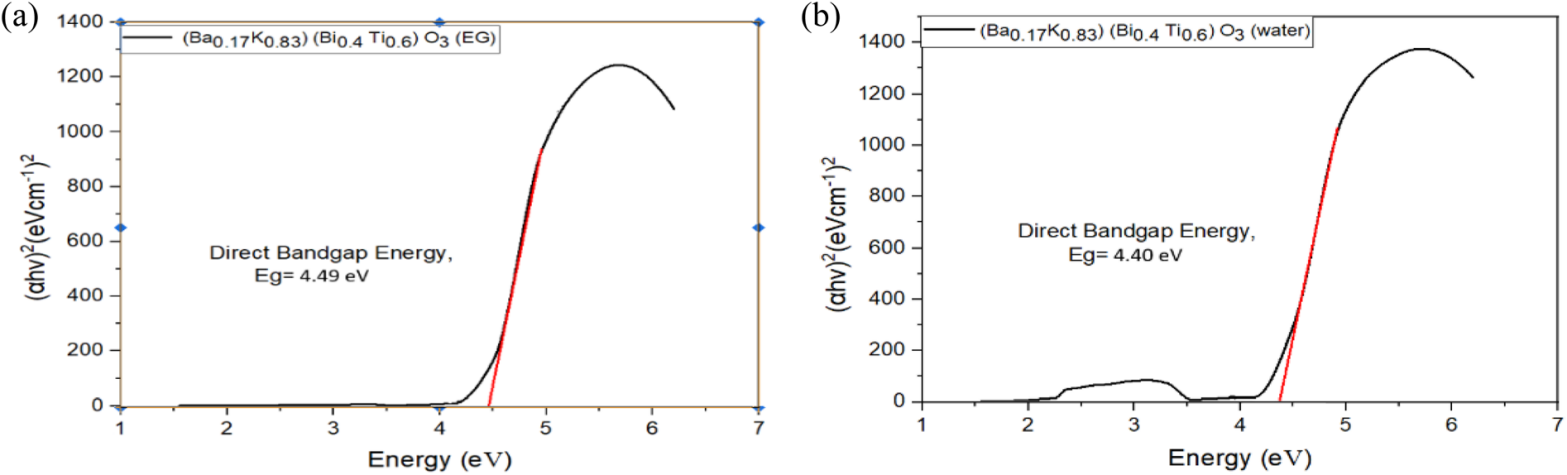
(a) Tauc plots of (Ba_0.17_K_0.83_)(Bi_0.4_Ti_0.6_)O_3_ prepared by ethylene glycol. (b) Tauc plots of (Ba_0.17_K_0.83_)(Bi_0.4_Ti_0.6_)O_3_ prepared by water.

Tauc plots for direct bandgap determination are presented in Fig. 11–13, where (*αhν*)^2^ is plotted against photon energy (*hν*) with the *y*-axis starting at zero to enable accurate extrapolation.^27^ This methodology, first proposed by Tauc in 1966, remains the standard approach for optical bandgap determination in semiconductors.^27^ Bandgap energies were determined by extrapolating the linear portion of the absorption edge (typically over 1–1.5 eV range) to the energy axis where (*αhν*)^2^ = 0. The intercept directly yields *E*_g_ without ambiguity. Corrected bandgap values are summarized in Table 8.

**Table 8:** Direct bandgap energy of synthesized [Ba_(1−*x*)_K_*x*_][Bi_(1−*y*)_Ti_*y*_]O_3_ samples^a^

Composition and solvent	Direct bandgap energy (eV)
(Ba_0.09_K_0.91_)(Bi_0.17_Ti_0.83_)O_3_ (EG)	4.52 ± 0.08
(Ba_0.09_K_0.91_)(Bi_0.17_Ti_0.83_)O_3_ (H_2_O)	4.50 ± 0.07
(Ba_0.2_K_0.8_)(Bi_0.2_Ti_0.8_)O_3_ (EG)	4.25 ± 0.06
(Ba_0.2_K_0.8_)(Bi_0.2_Ti_0.8_)O_3_ (H_2_O)	4.35 ± 0.07
(Ba_0.17_K_0.83_)(Bi_0.4_Ti_0.6_)O_3_ (EG)	4.49 ± 0.07
(Ba_0.17_K_0.83_)(Bi_0.4_Ti_0.6_)O_3_ (H_2_O)	4.40 ± 0.06

a Bandgap values determined from Tauc plot extrapolation using (*αhν*)^2^*vs. hν* plots for direct allowed transitions. Uncertainty represents 95% confidence interval from linear regression analysis of the Tauc plot linear region.

From Fig. 11–13 and Table 8, it is clearly seen that all the fabricated samples have large bandgaps exceeding 4.0 eV, classifying them as wide-bandgap semiconductors consistent with other Bi/Ti-based perovskites reported in literature.^12,19^ For comparison, BaTiO_3_ exhibits *E*_g_ ≈ 3.2 eV, pure BiFeO_3_ shows *E*_g_ ≈ 2.7 eV, and anatase TiO_2_ has *E*_g_ ≈ 3.2 eV.^12,19^ These large band gaps limit photocatalytic activity under ultraviolet light but are suitable for UV optoelectronic applications. The variation of bandgap across compositions (4.25 ± 0.06–4.52 ± 0.08 eV, Δ*E*_g_ = 0.34 eV) can be attributed to several factors:

(i) Compositional effects: increasing Bi content generally increases the bandgap due to the higher electronegativity of Bi^3+^ (2.02) compared to Ti^4+^ (1.54), which lowers the conduction band minimum energy.^12^ The valence band is primarily composed of O 2p orbitals hybridized with Bi 6s, while the conduction band derives from Bi 6p and Ti 3d orbitals.^19,27^

(ii) Crystal field effects: the degree of (Bi/Ti)O_6_ octahedral distortion varies with composition, affecting crystal field splitting of Ti 3d orbitals (t_2_g and eg levels) and thus the conduction band structure.^20,26^ Compositions near MPB regions may exhibit reduced bandgaps due to enhanced structural disorder and associated mid-gap states.

(iii) Defect chemistry: oxygen vacancies (V_O˙˙), common in hydrothermally synthesized oxides, introduce donor states approximately 0.5–1.0 eV below the conduction band, effectively reducing the optical bandgap.^12^ EG-synthesized samples generally show slightly different bandgaps compared to water-synthesized samples of identical composition, likely reflecting differences in V_O concentrations arising from distinct redox environments.^14^

(iv) Quantum confinement effects: the nanoscale crystallite sizes (Table 4, ranging 193–429 Å) may introduce weak quantum confinement, slightly increasing bandgaps compared to bulk materials according to Δ*E* ∝ 1/*d*^2^, where *d* is crystallite diameter.^12^ However, this effect becomes significant only for *d* < 10 nm (100 Å), suggesting minimal contribution in our samples.

Application relevance: the wide bandgap (>4 eV) makes these materials suitable for ultraviolet optoelectronic applications including UV photodetectors, transparent conducting oxides (when appropriately doped), frequency-conversion devices (second harmonic generation), and deep-UV light-emitting diodes.^12^ The systematic bandgap tunability through compositional control (Δ*E*_g_ ≈ 0.34 eV across the series) suggests potential for tailoring optical properties for specific applications. The unique combination of hexagonal symmetry and high thermal stability supports future device integration.

To contextualize the potential of [Ba_(1−*x*)_K_*x*_][Bi_(1−*y*)_Ti_*y*_]O_3_ for UV optoelectronic applications, Table 9 compares key parameters with established UV-active semiconductor materials. Our materials occupy the “deep-UV” spectral region (wavelengths < 280 nm, photon energies > 4.4 eV), positioning them between conventional UV-A absorbers (ZnO, GaN) and extreme deep-UV materials (AlN, diamond). This wavelength range is particularly relevant for applications in solar-blind UV photodetection, water/air purification *via* advanced oxidation, and next-generation UV lithography.

**Table 9:** Comparison with UV optoelectronic materials

Material	Bandgap (eV)	Absorption range	Synthesis *T* (°C)	Ref.
This work	4.25–4.52	Deep UV (275–283 nm)	220	This work
ZnO	3.37	UV-A (300–368 nm)	400–900	31
GaN	3.4	UV-A (300–365 nm)	>1000	32
BaTiO_3_	3.2	UV-A (310–388 nm)	900–1200	33
TiO_2_	3.0–3.2	UV-A (310–413 nm)	450–800	34
β-Ga_2_O_3_	4.6–4.9	Deep UV (253–270 nm)	800–1200	35
AlN	6.2	Deep UV (<200 nm)	>1200	36
Diamond	5.5	Deep UV (<225 nm)	>800 (CVD)	37

Our [Ba_(1−*x*)_K_*x*_][Bi_(1−*y*)_Ti_*y*_]O_3_ materials (*E*_g_ = 4.25–4.52 eV) occupy a strategic position in the deep-UV regime, comparable to β-Ga_2_O_3_ (*E*_g_ = 4.6–4.9 eV), which is emerging as a promising material for solar-blind UV photodetectors and high-power electronics. The key advantages of our materials include: (1) significantly lower synthesis temperature (220 °C *vs.* 800–1200 °C for β-Ga_2_O_3_, >1000 °C for GaN, >1200 °C for AlN), offering ∼75–85% energy reduction and compatibility with temperature-sensitive substrates; (2) compositional bandgap tunability (Δ*E*_g_ = 0.27 eV across the composition series), enabling wavelength-selective UV detection within the 275–292 nm range; (3) solution-based hydrothermal processing, which is more scalable and cost-effective than vapor-phase methods (CVD, MOCVD) required for GaN and AlN; (4) lead-free, environmentally benign composition, addressing toxicity concerns of some alternative UV materials. Current limitations include: (1) lack of demonstrated electrical properties – carrier concentration, mobility, and conductivity remain uncharacterized; (2) absence of optical quantum efficiency measurements; (3) no device-level validation (photodetector responsivity, detectivity, response time); (4) unknown radiation hardness and long-term stability under UV exposure. These represent critical next steps for practical application development. Nevertheless, the combination of deep-UV absorption, low-temperature synthesis, compositional flexibility, and structural stability establishes [Ba_(1−*x*)_K_*x*_][Bi_(1−*y*)_Ti_*y*_]O_3_ as a promising candidate material platform warranting further optoelectronic characterization and device fabrication studies.

### 3.7. Integrated structure–property relationships

The complementary characterization techniques provide comprehensive understanding of structure-property relationships. Correlating multi-technique data reveals key insights:

XRD-EDS correlation: the hexagonal structure (*P*6_3_/*m*) requires specific A/B-site occupancies for charge neutrality. Tolerance factor *t* = (*r*A + *r*O)/[√2(*r*B + *r*O)] ranges from 0.92 to 0.96, predicting stable perovskite formation consistent with XRD. EDS-confirmed ratios validate target stoichiometry and support Rietveld-refined site occupancies.

XRD-FTIR correlation: the crystallographic framework informs FTIR assignments. Metal–oxygen vibrations (Ti–O–Ti at 450–600 cm^−1^, Bi–O–Bi at 740–880 cm^−1^) correspond to specific octahedral environments. Peak intensity ratios correlate with compositional ratios from EDS.

XRD-TGA correlation: smaller EG crystallites (193–421 Å) exhibit greater surface area (∼15–30 m^2^ g^−1^), explaining larger mass losses (12–18%) *versus* water (3–7%). The TGA endotherm at ∼720 °C (α-Bi_2_O_3_ → δ-Bi_2_O_3_ transition) suggests Bi-rich surface segregation consistent with minor Bi_2_Ti_2_O_7_ pyrochlore detected by XRD.

XRD-UV-vis correlation: optical bandgap derives from crystal structure. Hexagonal D3d symmetry alters Ti 3d splitting differently than cubic Oh, contributing to larger bandgaps (4.25–4.52 eV) *versus* cubic BaTiO_3_ (3.2 eV). Bandgap increase with Bi content correlates with unit cell compression.

Solvent effect integration: all techniques reveal solvent-dependent differences: EG → lower crystallinity, smaller crystallites, hexagonal morphology, large mass loss; water → higher crystallinity, moderate crystallites, flake morphology, minimal mass loss. These link mechanistically to solvent viscosity affecting diffusion and growth kinetics.

This integrative analysis demonstrates coupled structural–compositional–thermal–optical properties systematically engineerable through composition and solvent selection.

## 4. Conclusion

K/Bi-doped perovskite materials [Ba_(1−*x*)_K_*x*_][Bi_(1−*y*)_Ti_*y*_]O_3_ were successfully synthesized *via* low-temperature hydrothermal methods at 220 °C, demonstrating systematic property control through solvent engineering. X-ray diffraction with Rietveld refinement confirmed hexagonal perovskite structures (space group *P*6_3_/*m*) with solvent-dependent crystallinity: water-based synthesis produced higher crystallinity (73–86%) and superior thermal stability (<5% mass loss to 800 °C), while ethylene glycol yielded distinct hexagonal faceted morphologies. UV-vis spectroscopy revealed wide optical bandgaps (4.25 ± 0.06 to 4.52 ± 0.08 eV) tunable through compositional control, positioning these materials for deep-UV optoelectronic applications including photodetectors and transparent conducting oxides. The hydrothermal approach offers ∼75% energy reduction *versus* conventional solid-state routes (>900 °C), reduced alkaline element volatility, and scalability to industrial autoclave systems. However, this study is limited to powder-phase characterization. Future work requires ceramic densification, direct ferroelectric characterization through P–E hysteresis and piezoelectric measurements, advanced defect analysis (XPS for oxidation state confirmation, EPR for defect identification), TEM/HRTEM/SAED for nanoscale structural confirmation, dielectric measurements (*ε*_r_*vs. T*, *ε*_r_*vs.* frequency) to assess potential ferroelectric behavior, and device fabrication to validate practical applications. Future thermal characterization should employ slower TGA heating rates (10 °C min^−1^) to better resolve decomposition steps. While XPS would provide complementary oxidation state validation, and TEM would confirm nanoscale crystallinity, the current multi-technique approach (XRD with Rietveld refinement, SEM-EDS, ICP-OES, CHN, FTIR, TGA, UV-vis) provides robust evidence for the reported perovskite formation. This work establishes an eco-friendly synthetic platform for lead-free quaternary perovskites with systematic property engineering through solvent selection, contributing to environmentally benign alternatives for UV optoelectronic materials.

## Author contributions

Md. Golam Sarowar: conceptualization, methodology, software, validation, formal analysis, investigation, resources, data curation, writing – original draft, visualization. Dr Mirza Humaun Kabir Rubel: writing – review & editing, data curation, supervision, project administration.

## Conflicts of interest

The authors declare that they have no known competing financial interests or personal relationships that could have appeared to influence the work reported in this paper.

## Data Availability

The data supporting the findings of this study are available from the corresponding author upon reasonable request to s1610378125@ru.ac.bd.
