# Correction: Solvent-mediated hydrothermal synthesis of K/Bi-doped [Ba_(1−*x*)_K_*x*_][Bi_(1−*y*)_Ti_*y*_]O_3_ perovskites: structure–property relationships and bandgap engineering

**DOI:** 10.1039/d6ra90096h

**Published:** 2026-08-11

**Authors:** Mahfuza Rahman Promy, Md. Golam Sarowar, Mirza Humaun Kabir Rubel

**Affiliations:** a Department of Materials Science and Engineering, University of Rajshahi Bangladesh mhk_mse@ru.ac.bd

## Abstract

Correction for ‘Solvent-mediated hydrothermal synthesis of K/Bi-doped [Ba_(1−*x*)_K_*x*_][Bi_(1−*y*)_Ti_*y*_]O_3_ perovskites: structure–property relationships and bandgap engineering’ by Md. Golam Sarowar *et al.*, *RSC Adv.*, 2026, **16**, 9890–9904, https://doi.org/10.1039/d6ra00023a.

The authors regret that the first author Mahfuza Rahman Promy was omitted from the list of authors of this work, and Md. Golam Sarowar was designated as the first author instead. Furthermore, the authors regret that Md. Golam Sarowar was incorrectly designated as the corresponding author instead of Mirza Hamaun Kabir Rubel. The correct authorship and contact details are as shown here.

The Royal Society of Chemistry apologises for these errors and any consequent inconvenience to authors and readers.

